# The effect of surface anisotropy in the slippery zone of *Nepenthes alata* pitchers on beetle attachment

**DOI:** 10.3762/bjnano.2.35

**Published:** 2011-06-16

**Authors:** Elena V Gorb, Stanislav N Gorb

**Affiliations:** 1 Department of Functional Morphology and Biomechanics, Zoological Institute, University of Kiel, Am Botanischen Garten 1–9, D-24098 Kiel, Germany

**Keywords:** adhesive pads, claws, *Coccinella septempunctata*, insect–plant interactions, traction force

## Abstract

The slippery zone in pitchers of the carnivorous plant *Nepenthes alata* bears scattered prominent lunate cells and displays continuous epicuticular crystalline wax coverage. The aim of this study was to examine the influence of the surface anisotropy, caused by the shape of lunate cells, on insect attachment ability. Traction tests with ladybird beetles *Coccinella septempunctata* were performed in two types of experiments, where surface samples of (1) intact pitchers, (2) chemically de-waxed pitchers, and (3) their polymer replicas were placed horizontally. Beetle traction forces were measured when they walked on test surfaces in either an upward (towards the peristome) or downward (towards the pitcher bottom) direction, corresponding to the upright or inverted positions of the pitcher. On intact pitcher surfaces covered with both lunate cells and wax crystals, experiments showed significantly higher forces in the direction towards the pitcher bottom. To distinguish between the contributions, from claw interlocking and pad adhesion, to insect attachment on the pitcher surfaces, intact versus claw-ablated beetles were used in the second type of experiment. On both de-waxed plant samples and their replicas, intact insects generated much higher forces in the downward direction compared to the upward one, whereas clawless insects did not. These results led to the conclusion that, (i) due to the particular shape of lunate cells, the pitcher surface has anisotropic properties in terms of insect attachment, and (ii) claws were mainly responsible for attachment enhancement in the downward pitcher direction, since, in this direction, they could interlock with overhanging edges of lunate cells.

## Introduction

Pitcher-shaped trapping organs produced at the tips of tendrils are characteristic for carnivorous plants from the genus *Nepenthes* [1–2]. These traps, using a passive pitfall mechanism for capturing, mainly, invertebrates, consist of several functional zones specialized for prey attraction, capture, retention, digestion, and uptake of nutrients [3–6]. During the past few decades, different aspects of *Nepenthes* biology, among them the structure and functions of pitchers, especially with respect to their trapping efficiency, have been the focus of numerous structural and experimental studies and field observations (review in [7]). For example, the importance of the pitcher rim, the peristome, for initial prey capture due to its specific microstructure combined with both surface hydrophilicity and secretion of hygroscopic nectar, was recently discovered [8–11]. In addition, the digestive fluid collected in the lower part of the pitcher was recently found to be highly viscous thus preventing trapped insects from escaping [12–13].

Although the slippery zone, situated inside the pitcher just below the peristome in the majority of *Nepenthes* species, was recognised long ago as an important structure for insect trapping and retention, due to its particular downward-directed lunate cells and thick crystalline wax coverage [14–16], the contribution of the microstructure to the anti-adhesive function of this surface is still being actively discussed. Numerous studies were recently performed to investigate the micromorphology, chemistry, properties of pitcher waxes, and their effect on insect attachment ability in several *Nepenthes* species (e.g., [17–26]). Using different experimental approaches, the authors explained the prevention of insect adhesion via contamination of adhesive pads by wax crystals and/or reduction of the real contact area caused by surface micro-roughness [19,22,25–26]. Also, due to the fragile and brittle nature of wax crystals and their small dimensions, insects cannot apply their claws for interlocking with crystals in order to climb up the pitcher wall [22,25–26].

As for the potential role of lunate cells in hindering insect attachment, the effect of surface anisotropy caused by these cells was postulated for the first time more than a century ago [15,27]. The authors suggested that these cells should be turned upside down to allow claw clinging by insects. However, the experiments performed by Knoll [15] could not show such an effect on insect locomotion. Whereas the morphology, distribution and origin of lunate cells, considered to be transformed stomatal guard cells, were later widely discussed in the literature [16–17,26,28–29], the experimental study to test the above hypothesis was only recently carried out. It was found that ants *Iridomyrmex humilis* moved faster and escaped more successfully when de-waxed pitchers of *N. alata* were inverted [18]. What still remains unclear is (1) the effect of the surface anisotropy of intact plant surfaces on insect attachment and (2) the contact mechanism responsible for this effect, although these issues have been repeatedly raised in discussions by recent authors (e.g., [25–26]).

This study was undertaken in order to directly examine the influence of the lunate cells causing surface anisotropy in the slippery zone on insect attachment force. We performed traction tests with ladybird beetles *Coccinella septempunctata* (L.) in two different experiments on (1) intact pitchers of *N. alata* Blanco, (2) de-waxed pitchers, and (3) their polymer replicas. Forces on tethered beetles were measured when they walked in the upward (towards the peristome) or downward (towards the pitcher bottom) directions corresponding to the upright or inverted positions of the pitcher. In order to distinguish between the contribution from claw interlocking and from adhesion using tarsal adhesive pads to insect attachment on these surfaces, the performances of intact and clawless (the claws were amputated) beetles were compared in the second experiment.

## Results

### Structure of test surfaces and insect attachment organs

**Figure 1:**
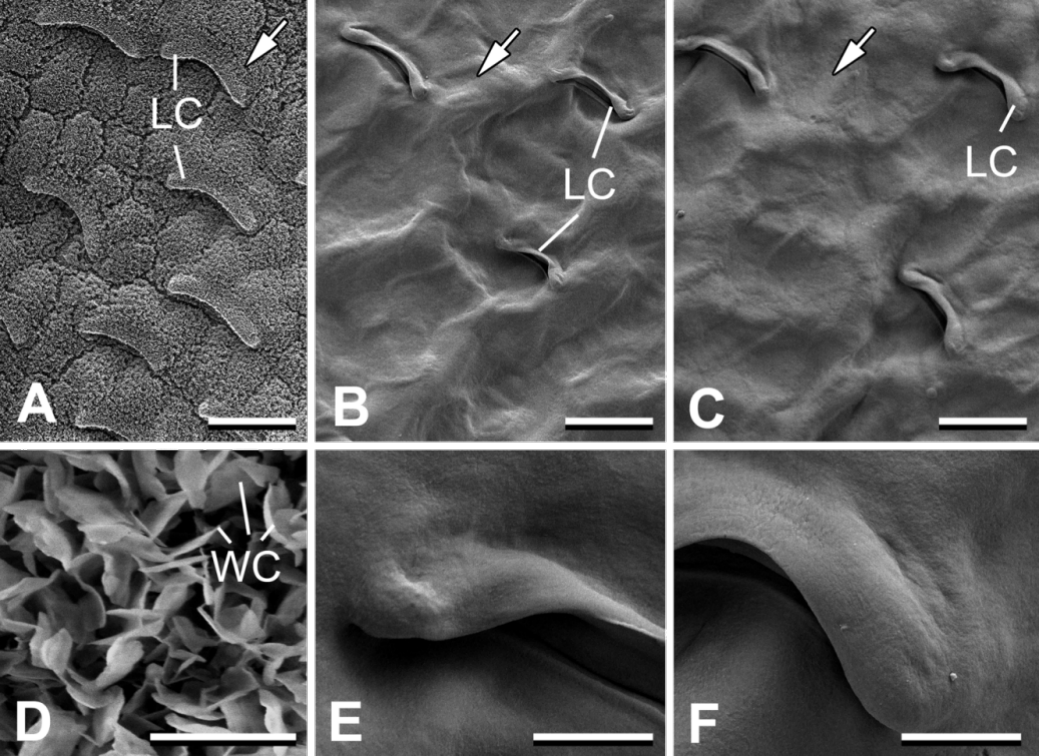
Cryo-SEM micrographs of intact *Nepenthes alata* pitchers (A, D) and SEM micrographs of de-waxed pitcher samples (B, E) and their polymer replicas (C, F) used for traction experiments. LC: lunate cells; WC: wax crystals. White arrows indicate direction to the pitcher bottom. Scale bars = 50 μm (A–C), 2 μm (D), 10 μm (E, F).

**Figure 2:**
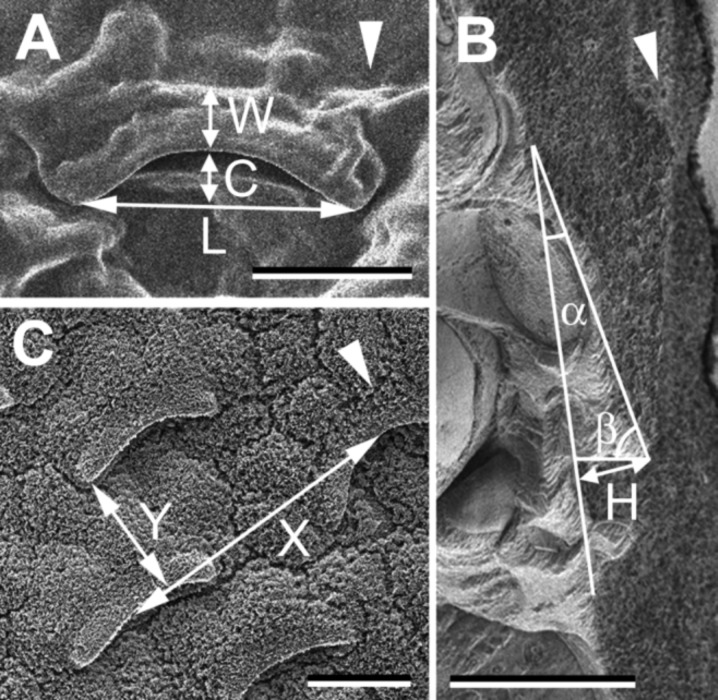
Morphometrical variables and distribution of lunate cells measured in SEM micrograph of a de-waxed surface (A) and cryo-SEM micrographs of an intact surface (B, C) of the slippery zone in *Nepenthes alata* pitchers: α = 19.53 ± 3.92°, β = 68.12 ± 17.01°, *C* = 6.95 ± 1.46 μm, *H* = 9.41 ± 2.08 μm, *L* = 35.51 ± 6.02 μm, *W* = 7.21 ± 1.01 μm, *X* = 84.90 ± 29.39 μm, *Y* = 51.16 ± 16.05 μm, N = 10–20. A, C: Top view of the surfaces. B: Fractured sample, used to measure variables α and β (angles between the exposed side of the lunate cell and its adjoining sides). White arrowheads indicate direction to the pitcher bottom. Scale bars = 20 μm. [B: courtesy of M. Benz (Department of Functional Morphology and Biomechanics, Zoological Institute, Christian Albrecht University of Kiel, Germany).]

The intact slippery zone of the *N. alata* pitcher bears relatively large, prominent lunate cells scattered between tabular epidermal cells and microscopic epicuticular wax crystals on top of both cell types (Figure 1A and Figure 1D). Numerous lunate cells (477.3 ± 46.02 per mm^2^, N = 3) are regularly distributed singly over the surface, whereas wax crystals form a continuous coverage. Lunate cells have a special crescent shape with their ends pointed toward the pitcher bottom (Figure 1A, Figure 2A and Figure 2C). The exposed upper side of the cell is sloped towards the pitcher mouth so that the lower cell margin becomes prominent and hangs over the adjoining cell (Figure 2B). Data on lunate cell dimensions and their distribution over the surface are given in Figure 2. These cells are responsible for the anisotropic surface relief of up to 10 μm in height, whereas crystalline wax coverage creates an additional roughness in the range of about 1 µm.

De-waxed pitcher samples and their polymer replicas displayed a very similar surface structure with a clear pattern of lunate cells (Figure 1B and Figure 1C). Wax crystals were completely removed by chloroform and the surface of lunate cells, as well as areas between them, became smooth (Figure 1E and Figure 1F). These samples additionally showed some waviness caused by the corrugation of tabular epidermal cells due to the drying process.

**Figure 3:**
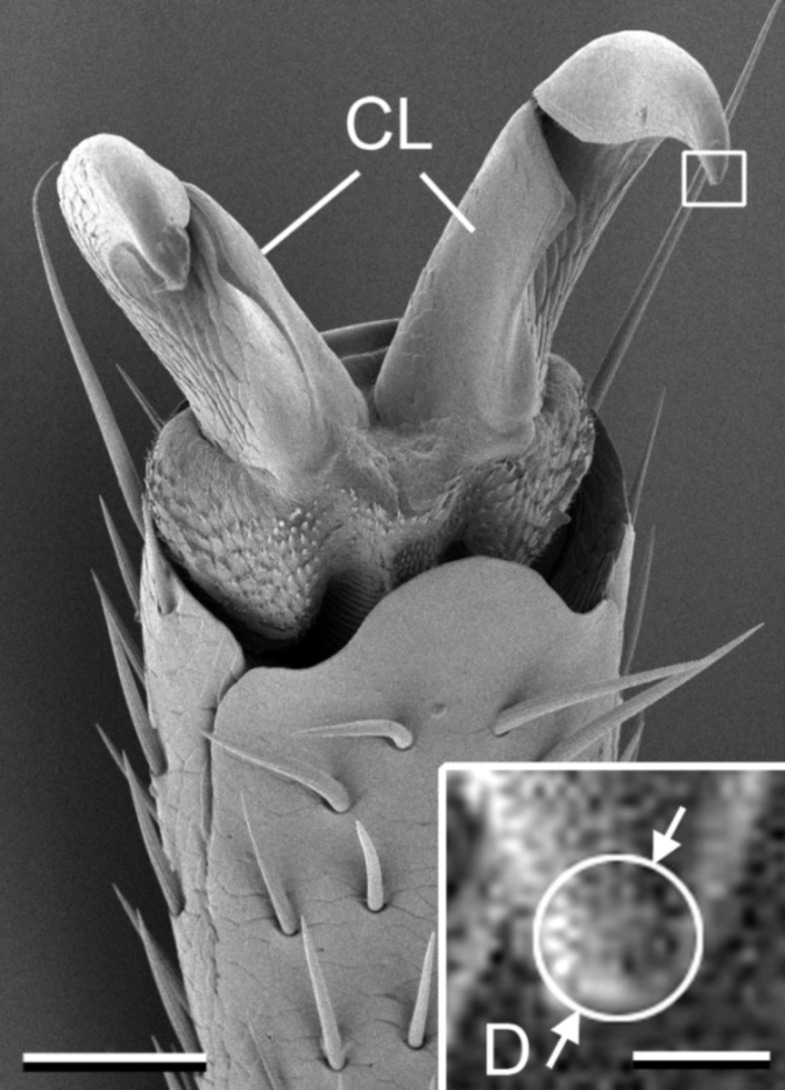
SEM micrographs of the distal part of the tarsus in the *Coccinella septempunctata* beetle. Inset shows magnified claw tip, where the white inscribed circle was used to measure the diameter of the claw tip. CL: claws; D: diameter of the claw tip. Scale bar = 50 μm. Scale bar of the inset = 5 μm.

The tarsus of the *C. septempunctata* beetle ends distally with two ventrally curved claws having tip diameters of 3.7 ± 0.64 μm (N = 10, Figure 3). Attachment pads belong to the hairy type of locomotory organs in insects. Pads and types of adhesive setae in this beetle species have been previously described in detail by [30].

### Traction forces of beetles on different surface samples

**Figure 4:**
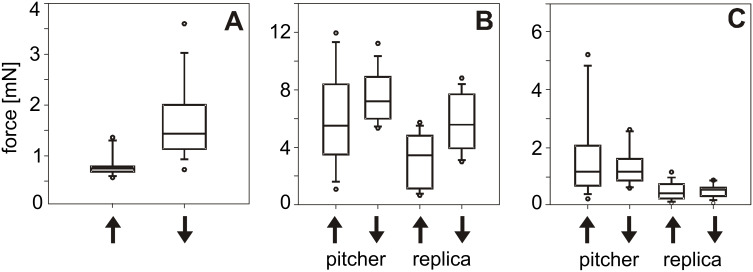
Results of traction force tests with *Coccinella septempunctata* beetles on different surfaces. (A) Experiments with both pitchers and insects intact. (B) Experiments with intact insects on de-waxed pitchers and their replicas. (C) Experiments with clawless insects on de-waxed pitchers and their replicas. Arrows pointing up indicate movement toward the pitcher peristome; arrows pointing down indicate movement towards the pitcher bottom.

On intact pitcher surfaces bearing both lunate cells and epicuticular crystalline waxes, traction forces of insects were drastically reduced compared to those measured on a glass sample (glass versus pitcher upward: t = 6.400; glass versus pitcher downward: t = 6.045, d.f*.* = 14, p < 0.001, paired t-test) (Figure 4A, Table 1). Beetles showed significantly lower force values when they moved in the upward direction compared to the downward one (upward versus downward: t = −4.271, d.f. = 14, p < 0.001, paired t-test).

**Table 1:** Traction forces (in mN) generated by *Coccinella septempunctata* beetles.

experiment	surface and run direction	mean value	st. dev.	N

both intact pitchers and beetles	glass	7.56	4.282	30
	intact pitcher upward	0.29	0.220	15
	intact pitcher downward	1.20	0.721	15

intact beetles on de-waxed pitchers and their replicas	glass	9.17	3.868	15
	de-waxed pitcher upward	5.78	3.252	15
	de-waxed pitcher downward	7.58	1.711	15
	replica upward	3.00	1.843	15
	replica downward	5.65	2.050	15

clawless beetles on de-waxed pitchers and their replicas	glass	2.27	1.854	15
	de-waxed pitcher upward	1.68	1.486	15
	de-waxed pitcher downward	1.29	0.614	15
	replica upward	0.51	0.315	15
	replica downward	0.51	0.229	15

In experiments with treated de-waxed pitchers and their replicas, insects both intact and clawless generated statistically different forces on tested surfaces, with forces on glass being higher and more variable than on other substrates (intact: H_4,74_ = 30.033; clawless: H_4,74_ = 38.003, p < 0.001, Kruskal–Wallis one way ANOVA on ranks) (Figure 4B and Figure 4C, Table 1).

Intact beetles exhibited high traction forces in the downward direction than in the upward one in both cases, on de-waxed pitchers and their replicas (de-waxed pitcher, upward versus downward: t = −2.327, p = 0.036; replica, upward versus downward: t = −3.060, p = 0.003, d.f. = 14, paired t-test) (Figure 4B, Table 1). In experiments with these beetles, forces on polymer replicas were lower compared to those on de-waxed pitchers (de-waxed pitcher upward versus replica upward: t = 4.006, p = 0.001; de-waxed pitcher downward versus replica downward: t = 2.834, p = 0.013, d.f. = 14, paired t-test).

Tests with clawless insects showed no significant differences in force values between upward and downward directions on either de-waxed plant samples or replicas (de-waxed pitcher, upward versus downward: W = −14.000, T+ = 53.000, T− = −67.000, p = 0.720, Wilcoxon signed rank test; replica, upward versus downward: t = 0.079, d.f. = 14, p = 0.938, paired t-test) (Figure 4C, Table 1). Just as in experiments with intact insects, clawless ones also produced lower forces on replicas than on de-waxed pitchers (de-waxed pitcher upward versus replica upward: W = −116.000, T+ = 2.000, T− = −118.000, p < 0.001, Wilcoxon signed rank test; de-waxed pitcher downward versus replica downward: t = 4.966, p < 0.001, d.f. = 14, paired t-test).

## Discussion

### Texture of natural and artificial pitcher surfaces

As in many other *Nepenthes* species, the slippery zone of *N. alata* represents a hierarchically structured surface created by the combination of several structural elements: Idioblasts (lunate cells) between tabular epidermal cells and superimposed crystalline wax coverage [31]. Pitchers lacking both lunate cells and wax were found only in a few species from this genus, such as *N. ampularia* [29], *N. bicalcarata* [8,16,32–35], *N. rafflesiana* [13,36], and *N. veitchii* [37].

The slippery zone in test pitchers displayed a surface morphology typical for *N. alata* and is described in detail in a series of recent studies [18,20,22,24–26]. Lunate cells in our pitcher samples were 1.5 times shorter than and half as wide as those reported in [26]. The heights of prominent margins of lunate cells were only 70% of those measured previously, whereas the density of cells exceeded twice the previously reported one. Here, we do not discuss the complex structure of the wax coverage. It is only important to note that the shapes, dimensions, and arrangement of uppermost wax crystals were in line with those previously observed.

De-waxed pitcher samples and their replicas had slightly corrugated surfaces with interspersed lunate cells, but still rather smooth at the micrometer scale. Similarly to previously reported data [18], no strong geometric artefacts in the pitcher surface, caused by the chloroform treatment, were found. Only some small changes in the increased waviness of the surface in de-waxed samples and their replicas were observed compared to the intact pitchers. Taking into account the relatively low height and aspect ratio of this waviness, it seemed to have no effect on the anisotropic properties of plant surfaces as discussed below.

### Anisotropy effect on insect attachment

Insect attachment ability in the slippery zone of the *N. alata* pitcher was previously studied in experiments designed to test the anti-adhesive effect of the wax coverage. It was shown in behavioural experiments that intact surfaces covered with wax crystals hindered the locomotion of ants *Iridomyrmex humilis*, as compared to de-waxed pitchers [18]. Traction experiments [22] demonstrated a significant reduction in the force generated by beetles *Adalia bipunctata* on two wax layers, as compared to both de-waxed pitchers and smooth hydrophilic glass used as a control. In slightly modified traction tests with locusts *Locusta migratoria*, forces on the pitcher surface were smaller than those on the smooth steel reference plate [26]. In all these studies, insects were tested walking in the upward direction of the pitcher.

Our present data on beetles walking in both upward and downward directions on the intact pitcher are consistent with the results of previous authors and also show drastically decreased traction force on wax-bearing surfaces compared to those measured on smooth glass. The decrease of insect attachment ability on the waxy pitcher surface can be explained by contamination of adhesive pads with wax crystals [1–2,19,22] and by reduction of the real contact area between the surface and insect attachment organs due to the surface micro-roughness [18,22,25–26]. A recently proposed model of the interactions of *N. alata* pitcher surface with insect pads and claws demonstrated that both stick insects *Carausius morosus* and ants *Lasius niger* can apply neither their pads nor claws for attachment to the crystalline wax surface with such a micro-roughness [25]. Due to the assumed mechanical stability of wax crystals, the hindering of insect attachment is explained solely by the reduction of the contact area caused by the surface micro-roughness. However, our previous theoretical study on the fracture behaviour of wax crystals, based on data on the crystal geometry in seven plant species and data on the mechanical properties of plant waxes, shows that crystals with very small cross sections and high slenderness ratios can break under the weight of middle-sized or even small-sized insects [38]. These results can also be applied to platelet-shaped crystals in *Nepenthes* plants.

Force measurements on insects running in different directions (upward versus downward) on the intact pitcher surface were performed here for the first time. In spite of the general large reduction of insect attachment ability on waxy surfaces, we detected a significant difference between forces generated in different directions: Beetles performed more strongly moving downwards than upwards. Similar results were obtained in tests with intact beetles on de-waxed pitchers and their polymer replicas, where force values were higher than on intact plant surfaces. These data concur with previous findings showing that ants *Iridomyrmex humilis* climbed up the surface much more successfully and faster on inverted de-waxed pitchers [18]*.* In unprecedented tests with clawless beetles, conducted to confirm experimentally the contribution made by claws in clinging to the pitcher surface, no differences between traction forces measured in different directions of the pitcher were found on either de-waxed pitchers or their replicas. This result indicates to us the principal role of claws in interlocking to inverted lunate cells. Furthermore, the slippery effect of the pitcher in the upright position is partially due to the failure of claw interlocking.

According to [39], successful attachment of an insect by claw interlocking is provided when the diameter of the claw tip is smaller than the size of surface asperities (or cavities). Since the height of overhanging edges in lunate cells (> 9 μm) exceeds the claw tip diameter (about 4 μm) in the tested beetle species, sufficient attachment of the beetle to the slippery zone should occur. This effect was observed in the present study in the experiments with intact insects on inverted surfaces, where inverted lunate cells served as anchorage sites for claws. Moving in an upward direction, as in a natural situation where an insect tries to escape from the pitcher, the beetles were unable to employ their claws, because of the downward orientation of the overhanging cell edges. Thus, the anisotropic morphology of the lunate cells causes the anisotropic tribological properties of the slippery pitcher zone. The effect of cell shape on the prevention of claw anchorage has also been previously reported for another pitcher surface, the peristome [8], where insect locomotion is also promoted in the downward pitcher direction and prevented in the upward one.

Since de-waxed pitchers and polymer replicas showed very similar topographies, better performance of beetles on de-waxed pitcher surfaces than on their replicas may be explained by the different mechanical and physicochemical properties of both substrates. We surmise that the softer and more compliant material of the plant tissue promotes insect interlocking due to easier indentation of claw tips into a softer substrate. This effect is reinforced by the higher surface energy of de-waxed plant samples (compared with epoxy replicas) presumably leading to some increase in pad adhesion. In an experimental study on frictional properties of the adhesive pads of locusts *Oedaleus infernalis* on several artificial substrates having similar roughness but different stiffness, lower insect attachment ability on zinc plates (stiff) compared to polyvinyl chloride substrates (soft) was also demonstrated [40].

Based on the data presented here, we may conclude that, due to the particular shape of the lunate cells, the slippery pitcher zone has anisotropic frictional properties in terms of insect attachment. Claws are primarily responsible for attachment enhancement on inverted pitchers, since they could interlock with the overhanging edges of the lunate cells. Due to the failure of the claw interlocking on upright pitchers, insects can slide more easily inside the pitcher. In intact pitchers, the latter effect is reinforced by a strong reduction of adhesion by adhesive pads due to the crystalline wax coverage.

### Outlook: biomimetic potential and implementation

The anisotropic properties of the slippery zone in *N. alata* pitchers and their polymer replicas make these surfaces suitable as possible prototype materials for technical implementations requiring frictional anisotropy. Possible applications range from braking systems of cars to haptic security signs in banknotes. There is only very limited information on the anisotropic tribological properties of natural surfaces in general, and the present paper opens a new field in the biomechanics and biomimetics of this kind of system. Here, we were able to establish the first artificial prototypes of anisotropic surface structures by a two-step replicating process. In the framework of a joint project within the DFG priority program SPP 1420, whose members include the Department of Functional Morphology and Biomechanics at the University of Kiel and the Institute for Chemistry at the University of Osnabrück, the hierarchical structure of the slippery zone is currently being analysed at different levels of organization, in order to use estimated geometrical variables to mimic the surface in technical materials using various available micro- and nanofabrication technologies. The moulding technique applied here has clearly demonstrated that the structural anisotropy of a certain dimension affects the frictional anisotropy (at least for crawling insects). However, the implementation of such anisotropy in industrial polymer foils, especially on a large production scale, still remains a rather challenging task.

## Experimental

### Plant and insect species

Mature upper pitchers of the tropical pitcher plant *N. alata* were harvested from plants grown in the greenhouse of the Botanical Garden at the University of Hohenheim (Stuttgart, Germany) and kept in plastic bags in a refrigerator for the duration of the experiments.

Seven-spotted ladybird beetles *C. septempunctata* were used in traction experiments because of their appropriate size (6–8 mm in body length), availability, and robustness in traction experiments. The capture of coleopteran insects by pitchers of several *Nepenthes* species has been previously reported [3–4,6]. Adult beetles were collected from plants along roadsides near Kronshagen (Germany). They were kept in small ventilated cages at a temperature of 22–24 °C and relative humidity of 60–75% for four days and fed with a weak solution of honey in tap water. Water was changed daily and the cages were sprayed with water twice a day.

### Test surfaces

Three types of substrates were used for traction tests with insects: (1) intact slippery zone of *N. alata* pitchers, (2) de-waxed slippery zone, and (3) polymer replicas of the de-waxed slippery zone.

Three strips, each 7–8 cm long and 1.5–2 cm wide, were cut with a razor blade from the slippery zone of three pitchers from three *Nepenthes* plants and directly used in the first type of experiments. Three other similar strips, cut from the same pitchers, were rinsed for 20 s in warm chloroform to remove the crystalline wax coverage from the surface, air dried and used as (i) de-waxed pitcher samples and (ii) for making high-resolution polymer replicas of this surface. Replicas were additionally used because treatment of plant surfaces with chloroform leads to the removal not only of epicuticular waxes, but also intracuticular ones and therefore changes the chemical and physical properties of the plant surface [21]. To eliminate these effects on insect attachment and examine the contribution of surface topography, we prepared replicas of the de-waxed pitchers and tested them together with de-waxed plant samples in the second type of traction experiments. Replicas were obtained by applying the two-component dental wax (Coltène President light body, Coltène Whaledent Dentalvertriebs Ltd., Konstanz, Germany) and Spurr resin [41] according to the two-step moulding method [42].

### Microscopy

The microstructure of the above described intact and de-waxed pitcher samples was studied by scanning electron microscope (Hitachi S-4800, Hitachi High-Technologies Corporation, Tokyo, Japan) equipped with a cryo-preparation system (Gatan ALTO 2500 cryo-preparation system, Gatan Inc., Abingdon, UK). The cryo-SEM method employed for studying intact pitcher samples was previously introduced in detail [24]. De-waxed pitcher samples and their polymer replicas were examined using a conventional SEM method as described in [22]. Morphometrical variables of the surface structures, such as lunate cell width *W*, length *L*, and height *C* of the span in the pendent cell margin, height *H* of the prominent cell margin relative to the adjoining cell, slopes of two free cell sides α and β*,* as well as intervals between lunate cells in vertical *Y* and side *X* directions (Figure 2) were measured from digital images using SigmaScan Pro 5 software (SPSS Inc., Chicago, USA). Additionally, the density of lunate cells on the surface was estimated.

The attachment organs of beetles were studied in the SEM at an acceleration voltage of 3 kV. For this purpose, insects were air-dried, mounted with their dorsal side to aluminium holders, sputter-coated with gold-palladium (thickness 6 nm), and examined in SEM, as described above. The diameter of the claw tip (*D*) was estimated from digital images (Figure 3) using SigmaScan Pro 5 software according to [39].

### Traction experiments

Traction force measurements were performed with both male and female beetles (see [30] for a detailed method description). Two types of experiments were carried out with tethered beetles on horizontally placed substrates. Firstly, traction forces were measured on intact pitcher surfaces while intact insects walked either upwards (towards the peristome) or downwards (towards the pitcher bottom) corresponding to the upright or inverted positions of the pitcher. The test on a smooth hydrophilic glass surface, used as a reference substrate, was carried out prior to each test on the plant surface, in order to eliminate disabled insects and select only strong healthy ones for experiments. To exclude the effect of possible contamination of insect attachment organs by wax crystals on the force measured, the second experiment on the pitcher surface (in the opposite pitcher direction) was executed after the beetles had recovered overnight from the first experiment performed on the pitcher surface.

In the second type of experiment, we compared the performances of intact insects with those of insects having amputated claws. With each individual insect, two sets of tests were performed on the following, horizontally placed, substrates: (1) Smooth solid glass plate; (2) de-waxed pitcher surface in the upward (towards the peristome) running direction; (3) de-waxed pitcher surface in the downward (towards the pitcher bottom) running direction; (4) polymer replica of the de-waxed pitcher surface in the upward running direction; (5) polymer replica of the de-waxed pitcher surface in the downward running direction.

Tests on a glass surface were always conducted first for the same reason as in the first type of experiment. The use of the other surface samples was randomized to avoid any systematic artefacts due to testing the surfaces in a given order. At first, we tested intact insects on the five substrates mentioned above. The claws were then amputated and, after overnight recovery, the same insects were used in the second set of experiments. For claw amputation, the beetles were narcotised with CO_2_ for about 1 min, and then the claws were excised with small scissors.

The force-time curves obtained were used to calculate the maximal traction force of individual beetles. Tests were performed at a room temperature of 22–24 °C and 55–60% relative humidity. Fifteen beetles were tested in each type of experiment. In all, 210 force measurements were carried out.

Data are presented in the text as a mean value ± standard deviation. Kruskal-Wallis one way ANOVA on ranks was used to evaluate the differences in traction force values between substrate samples (software SigmaStat^®^ 3.1.1, Systat Software Inc., Richmond, California, USA). To compare forces between upward and downward running directions and between de-waxed pitchers and replicas, data were statistically analysed using paired t-test and Wilcoxon signed rank test (software SigmaStat^®^ 3.1.1).

## References

[1] Juniper B E, Burras J K (1962). New Sci.

[2] Juniper B E, Robins R J, Joel D M (1989). The Carnivorous Plants.

[3] Kato M, Hotta M, Tamin R, Itino I (1993). Trop Zool.

[4] Moran J A J (1996). Ecol.

[5] Adam J H (1997). Pertanika J Trop Agric Sci.

[6] Moran J A, Booth W E, Charles J K (1999). Ann Bot (Oxford, U K).

[7] Moran J A, Clarke C M (2010). Plant Signal Behav.

[8] Bohn H F, Federle W (2004). Proc Natl Acad Sci U S A.

[9] Bauer U, Bohn H F, Federle W (2008). Proc R Soc London, Ser B.

[10] Bauer U, Willmes C, Federle W (2009). Ann Bot (Oxford, U K).

[11] Bauer U, Federle W (2009). Plant Signal Behav.

[12] Gaume L, Forterre Y (2007). PLoS One.

[13] Gaume L, Di Gusto B (2009). Ann Bot (Oxford, U K).

[14] Macfarlane J M (1893). Ann Bot (Oxford, U K).

[15] Knoll F (1914). Jahrb Wiss Bot.

[16] Lloyd F E (1942). The Carnivorous Plants.

[17] Owen T P, Lennon K A (1999). Am J Bot.

[18] Gaume L, Gorb S, Rowe N (2002). New Phytol.

[19] Gaume L, Perret P, Gorb E, Gorb S, Labat J-J, Rowe N (2004). Arthropod Struct Dev.

[20] Riedel M, Eichner A, Jetter R (2003). Planta.

[21] Riedel M, Eichner A, Meimberg H, Jetter R (2007). Planta.

[22] Gorb E, Haas K, Henrich A, Enders S, Barbakadze N, Gorb S J (2005). Exp Biol.

[23] Gorb E V, Gorb S N (2006). Plant Biol (Hoboken, NJ, U S).

[24] Gorb E V, Gorb S N, Gorb S N (2009). Functional surfaces in the pitchers of the carnivorous plant Nepenthes alata: A cryo-SEM approach. Functional Surfaces in Biology.

[25] Scholz I, Bueckins M, Dolge L, Erlinghagen T, Weth A, Hischen F, Mayer J, Hoffmann S, Riederer M, Riedel M (2010). Exp Biol.

[26] Wang L, Zhou Q (2010). Adv Nat Sci.

[27] Bobisut O (1910). Sitzungsber Akad Wiss Wien, Math-Naturwiss Kl, Abt 1.

[28] Adams R M, Smith G W (1977). Am J Bot.

[29] Pant D D, Bhatnagar S (1977). Phytomorphology.

[30] Gorb E, Hosoda N, Miksch C, Gorb S J R (2010). Soc, Interface.

[31] Poppinga S, Koch K, Bohn H F, Barthlott W (2010). Funct Plant Biol.

[32] Clarke C M, Kitching R L J (1995). Trop Ecol.

[33] Cheek M, Jebb M (2001). Flora Malesiana. Series I – Seed Plants.

[34] Merbach M A, Zizka G, Fiala B, Maschwitz U, Booth W E (2001). Flora (Jena).

[35] Moran J A, Hawkins B J, Gowen B, Robbins S (2010). J Exp Bot.

[36] Di Gusto B, Gueroult M, Rowe N, Gaume L, Gorb S N (2009). The waxy surfaces in Nepenthes pitcher plants: Variability, adaptive significance and developmental evolution. Functional Surfaces in Biology.

[R37] 37Benz, M. J. Comparative study of the ultrastructure of the slippery zone in nine carnivorous *Nepenthes* species. Diploma Thesis, University of Stuttgart, Germany, 2009.

[38] Borodich F M, Gorb E V, Gorb S N (2010). Appl Phys A.

[39] Dai Z, Gorb S N, Schwarz U J (2002). Exp Biol.

[40] Wang L, Zhou Q, Xu S, Niu H (2009). Chin Sci Bull.

[41] Spurr A R J (1969). Ultrastruct Res.

[42] Gorb S N (2007). Microsc Today.

